# Re-analysis of the larval testis data on meiotic sex chromosome inactivation revealed evidence for tissue-specific gene expression related to the drosophila X chromosome

**DOI:** 10.1186/1741-7007-10-49

**Published:** 2012-06-12

**Authors:** Maria D Vibranovski, Yong E Zhang, Claus Kemkemer, Hedibert F Lopes, Timothy L Karr, Manyuan Long

**Affiliations:** 1 Department of Ecology and Evolution, The University of Chicago, 1101 East 57th Street, Chicago, IL 60637, USA; 2 Key Laboratory of the Zoological Systematics and Evolution, Institute of Zoology, Chinese Academy of Sciences, Beichen West Road, Chaoyang District, Beijing 100101, PR China; 3 The University of Chicago Booth School of Business, 5807 South Woodlawn Ave., Chicago, IL 60637, USA; 4 Center for Evolutionary Medicine and Informatics and Center for Infectious Diseases and Vaccinology, The Biodesign Institute, Arizona State University, 1001 South McAllister Ave., Tempe, AZ 85287, USA

## Abstract

**Background:**

Meiotic sex chromosome inactivation (MSCI) during spermatogenesis has been proposed as one of the evolutionary driving forces behind both the under-representation of male-biased genes on, and the gene movement out of, the X chromosome in *Drosophila*. However, the relevance of MSCI in shaping sex chromosome evolution is controversial. Here we examine two aspects of a recent study on testis gene expression (Mikhaylova and Nurminsky, *BMC Biol *2011, **9:**29) that failed to support the MSCI in *Drosophila*. First, Mikhaylova and Nurminsky found no differences between X-linked and autosomal genes based on the transcriptional profiling of the early testis development, and thus concluded that MSCI does not occur in *D. melanogaster*. Second, they also analyzed expression data from several *D. melanogaster *tissues and concluded that under-representation on the X chromosome is not an exclusive property of testis-biased genes, but instead, a general property of tissue-specific genes.

**Results:**

By re-analyzing the Mikhaylova and Nurminsky's testis data and the expression data on several *D. melanogaster *tissues, we made two major findings that refuted their original claims. First, the developmental testis data has generally greater experimental error than conventional analyses, which reduced significantly the power to detect chromosomal differences in expression. Nevertheless, our re-analysis observed significantly lower expression of the X chromosome in the genomic transcriptomes of later development stages of the testis, which is consistent with the MSCI hypothesis. Second, tissue-specific genes are also in general enriched with genes more expressed in testes than in ovaries, that is testis-biased genes. By completely excluding from the analyses the testis-biased genes, which are known to be under-represented in the X, we found that all the other tissue-specific genes are randomly distributed between the X chromosome and the autosomes.

**Conclusions:**

Our findings negate the original study of Mikhaylova and Nurminsky, which concluded a lack of MSCI and generalized the pattern of paucity in the X chromosome for tissue-specific genes in *Drosophila*. Therefore, MSCI and other selection-based models such as sexual antagonism, dosage compensation, and meiotic-drive continue to be viable models as driving forces shaping the genomic distribution of male-related genes in *Drosophila*.

## Background

It has been found that sex-biased genes, those more expressed in one sex than in the other, are not randomly distributed on the chromosomes in *Drosophila *[1-3]. Male-biased genes are generally under-represented on the X chromosome, except the very young genes, whereas female-biased genes are enriched on the X [1,3]. In addition, there is an excess of gene movement from the X chromosome to autosomal locations, with new retrogenes acquiring testis-biased expression pattern [2]. Those two related phenomena have been broadly observed in the *Drosophila *genus [4-9], in mosquitos [10-12], and mammals [13,14]. The X chromosomes from all *Drosophila *species analyzed, including Neo-X chromosomes, were found to be under-represented with male-biased genes [4,5]. Further, the excess movement of retrogenes and DNA-based duplications off the X chromosome was observed in 12 *Drosophila *species whose genomes were sequenced [8,9]. In *Drosophila*, gene movement off the X chromosome was suggested to be a mechanism by which the autosomes become enriched with male-biased genes [5].

These observations raise interesting questions about the processes shaping sex chromosome evolution, particularly the relationship between male-biased gene expression and the under-representation of this class of genes on the X chromosome. Over the past decade, four hypotheses, including sexual antagonism, meiotic sex chromosome inactivation, dosage compensation, and meiotic drive, have been proposed to interpret the paucity of male-biased X-linked genes [2,15-23]. The first hypothesis, sexual antagonism, assumes that sexually antagonistic forces drive male-biased expression. In such case, the X chromosome, which is present in a single copy in males compared to two copies in females, would have less opportunity to accumulate male-biased genes [15,16,21]. More specifically, sexually antagonistic dominant mutations with male-beneficial and female-detrimental effects have a higher probability of fixation on the autosomes [15,16]. However, a recent study has shown that sexual antagonistic genes tend to be preferentially located on the X chromosome [17]. This result suggests that sex-biased genes are not currently under sexual antagonistic selection but rather represent the partial or total resolution of the phenomenon [17]. The second hypothesis, dosage compensation, predicts that the hypertranscription of the X chromosome in *Drosophila *could further limit the up-regulation of genes and therefore prevent the origination of male-biased genes on the X chromosome [18,19]. The third hypothesis proposes that meiotic sex chromosome inactivation (MSCI) could favour the accumulation of testis-biased genes in the autosomes [2,20]. Different from X-linked genes, autosomal genes are free from the inactivation process and therefore have an increased probability of being expressed in males [2,20]. In the fourth hypothesis, meiotic drive alleles located on X chromosome and expressed during spermatogenesis could favour the evolution of autosomal male-biased genes as their potential suppressors [22,23].

Empirical evidence exists in support of most of these hypotheses suggesting that all of them may have played a role in chromosomal distribution of male-biased genes [1,18,19,24,25]. Evidence supporting the sexual antagonism hypothesis comes from the observation of the paucity of X-linked male-biased genes expressed in somatic tissues which do not undergo X chromosome inactivation [1], whereas evidence supporting the dosage compensation hypothesis comes from studies showing that: (1) male-biased genes are less likely to be bound by the MSL complex [19]; and (2) highly expressed male-biased genes are more rarely found on the X chromosome [18].

MSCI has been shown to occur in a wide range of taxa: mammals, nematodes, chicken, and *Drosophila *[20,24-29]. Although there is unequivocal evidence for MSCI in mammals, until recently the only indirect evidence for MCSI in *Drosophila *was from the pioneering work of Lifschytz and Lindsley [20]. There are now two major lines of supporting evidence for MSCI in *Drosophila *[24,25,29,30]. First, insertion into the X chromosome of genes carrying a testis-specific promoter had reduced expression compared to the same insertions into autosomes [24], a result consistent with the MSCI model. These results were further confirmed by a more exhaustive study of insertions across different regions of the entire X chromosome [29]. Second, a global analysis of gene expression between testis samples enriched with mitotic and meiotic cells showed a significant down-regulation of the X chromosome in agreement with MSCI [25]. Yet, a recent study argues that this X chromosome-specific down-regulation starts in earlier stages of the mitotic male germline [31].

Nonetheless, MSCI was demonstrated to be one of the driving forces for the genomic relocation of testis-biased genes [25]. First, the under-representation of testis-biased genes was found for genes over-expressed in meiosis, but not in mitosis [25]. Second, parent-retrogene pairs moving out of the X chromosome have higher complementary expression in meiosis, that is parental gene down-regulation and retrogene up-regulation, than those pairs moving between autosomes [25]. Those observations directly link the testis-biased X chromosome deficiency to a meiotic event as expected by MSCI in males.

However, a recent study using an alternative approach to assess MSCI in *Drosophila *claimed that there was no sign of X inactivation during male meiosis [32]. Different larval development stages were used to obtain testis with differing amounts of spermatogenic meiotic cells [32]. No differential expression between autosomes and X chromosomes was detected during larval development and therefore the global X inactivation in male germline was ruled out as a possible process [32]. The same study [32], using the public *Drosophila *expression dataset [33] analyzed the chromosomal distribution of tissue-specific genes and found that several non-sex-related tissues, besides the testis as previously thought [1,3-5], had paucity of X-linked genes. Taken together, the authors suggested that there was no evidence for MSCI and therefore could not be a driving force behind the chromosomal distribution of male-biased genes [32].

To better understand the difference between these analyses and previous conclusions, we re-analyzed the data of this recently published study [32]. First, we found that the larval testis data generated by Mikhaylova and Nurminsky [32] have low within-replicate correlations, which should make the detection of differential chromosomal expression practically impossible. Second, we also found that the tissue-specific gene datasets used by Mikhaylova and Nurminsky [32] were actually enriched with testis-biased genes. Using a non-enriched dataset after filtering out the testis-specific genes, we found that non-sex-biased tissue-specific genes were not under-represented on the X chromosome. In the sections below, we report the details of our re-analyses.

## Results

### Testis development expression of X-linked and autosomal testis-biased genes

Mikhaylova and Nurminsky [32] presented an alternative way to test MSCI in *Drosophila*. Instead of measuring the entire testis expression of adult flies with X-linked transgenes [24,29] or comparing the transcriptome of adult spermatogenic phases [25,30], they analyzed the expression profile of second and third larval testes [32]. During these stages, each single gonial cell, generated by the division of a stem cell every 10 h, is followed by four mitotic and two meiotic divisions. However, because the entire process of spermatogenesis requires approximately 250 h, postmeiotic processes and the production of mature sperm occur primarily during pupal and adult stages [34].

Mikhaylova and Nurminsky [32] took advantage of the spermatogenesis timeline and obtained RNA from the first wave of germline differentiation by dissecting larval testes collected from days 4 to 7 (second-instar larvae and the point in which the third-instar start to pupate, respectively). The meiotic divisions approximately occur at the beginning of pupation whereas the bulk of spermatid elongation occurs during the pupal stages [32,34]. Their designed experimental approach could be a useful system for examining MSCI because the number of somatic cells and spermatogonia is constant at all stages of larval development [32]. The number of spermatocytes, however, increases with time, becoming the majority of germ-line cell type present in the third-instar larvae [20,32,34]. Therefore, they reasoned that during later phases of development significant expression differences between chromosomes in meiotic phases should exist in the testis transcriptome as the spermatocytes accumulate and become mature [32].

**Figure 1:**
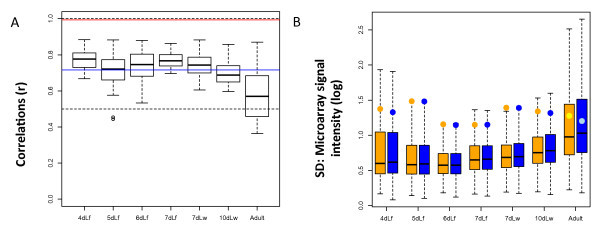
**Statistics for developing testis analyses**. Description of data analysis used in [32]. (**A**) Box plot of correlations within 10 replicates of each testis development stage. X-axis in (A and B) correspond to microarray experiments performed with testes isolated from either feeding (f) or wandering (w) larva (L) grown for 4, 5, 6, 7, or 10 days at 18°C. Adult flies correspond to 12-15 days of growth [32]. Dashed lines correspond to correlations equal to 0.5 and 1.0; the blue line is the overall average of all correlations (0.72); and the red line shows the minimal correlation found in [25]. (**B**) Box plot representing the distributions of the standard deviations of 10 replicates for approximately *n *= 14,000 *Drosophila *genes (transcripts) in each development stage. Note that the ratio between the standard deviation among genes (orange and blue circles in (B)) and the mean of the standard deviations within replicates of the same gene can be as low as 1.1. X-linked and autosomal statistics are shown in orange and blue, respectively.

In their first analysis, the expression profile along different development phases of eight X-linked and 18 autosomal testis-biased genes were measured by RT-PCR [32]. The MSCI model predicts no increase in expression (activation) for X-linked genes during the meiotic phases. The first argument against MSCI used by Mikhaylova and Nurminsky [32] is the observation of a dramatic increase in both X-linked and autosomal gene expression (Figure 1A and 1B in [32]). One key statistical treatment in this experiment is that they normalized their expression data using the expression from *rp49*, also known as *RpL32*, along the same developmental stages. It is known, however, that the expression profile of *rp49 *decreases from the first instar larvae to the pupae stage [35] (Additional File 1, Figure S1). This decrease was not taken into account in their analysis, consequently leading to an overestimation of the expression levels of the genes tested.

The second argument against MSCI used by Mikhaylova and Nurminsky [32] is the 'striking similar patterns of expression' shown by expression profiling of X-linked and autosomal genes in the meiotic germline (data presented in Figure 1C in [32]). This is in contrast to the MSCI hypothesis, which predicts significantly lower X-linked gene expression compared to autosomal genes. However, and in contrast to their conclusions, visual inspection of the expression levels shown in their Figure 1C [32] does show reduced expression of X-linked genes compared to autosomal genes throughout larval development. Unfortunately, it might be that the small number of the genes chosen for the RT-PCR experiment has large expression variation, which decreased the power to test between-chromosomal differences.

### Large experimental errors compromise statistical power to detect expression differences

In a second analysis, the authors examined the genome-wide X- and autosomal-linked gene expression during larval testis development (Figure 2 in [32]). They concluded from a visual inspection (without supporting statistical analysis of the data) that there was no observable reduction in X-linked gene expression and therefore MSCI does not occur in *Drosophila *[32]. This analysis and conclusions led us to further examine the statistical details of the normalized datasets used (available from ArrayExpress submission E-MEXP-1980 from [32] and here in the Additional file 2).

We investigated the quality and the reproducibility of the expression data used in the study. We plotted the correlation values obtained from replicate runs to assess reproducibility of the microarrays (Figure 1A). The pairwise correlations between 10 biological replicates of the same developmental phase are consistently lower (average 0.7; range 0.6-0.8) than expected in a quality microarray experiment [36]: usually higher than 0.9, suggesting a substantially greater experiment error within the replicates. The low correlation within experimental replicates suggests that the testis development expression data produced by Mikhaylova and Nurminsky [32] was significantly compromised by large experimental error, that is error between different expression measurements of the same gene in the same developmental phase.

To confirm this possibility, we calculated which portion of the experimental variability corresponds to the experimental error (Table 1). We found that the experimental error accounts for 25% to 45% of the variance. Mikhaylova and Nurminsky [32] have concluded for lack of global MSCI through the visual comparison of the distributions of gene expression for the autosomes and for the X chromosome. However, each gene expression was obtained by averaging the replicate measurements, which we found to have large experimental error. Therefore, those errors could significantly affect the comparisons between the average values of X- and autosomal-linked gene expression, decreasing the statistical power to detect any signal of MSCI.

**Table 1:** Variability of the experimental error

Days of larval developmental	4thF	5thF	6thF	7thF	7thW	10thW	Adult
**Experimental error (%)**	29.7%	33.3%	24.9%	23.9%	26.5%	32.2%	44.2%

Table describes the source (percentage) of the total variation that corresponds to the experimental error, that is, the measurement error within replicates of the same gene. Abbreviations for days and feeding stages follow Figure 1 legend.

We evaluated the effect of a large experimental error on the ability to detect MSCI by computing the variability within replicates of the same gene and calculating the standard deviations within replicates. We compared the distributions of those standard deviations (boxplot in Figure 1B) to the variability among genes, that is standard deviations of the means of chromosome expression (orange and blue circles in Figure 1B). We found that the latter is just slightly higher than the former, which means that the variation among genes is slight higher than the variation within the replicates of the same genes (lowest ratio is found for adults samples: 1.1). The effect of experimental errors can be individually noticed from their gene expression measurements, which were presented as log2-based [32], and therefore a unit difference corresponds to approximately a two-fold difference in signal intensity. Thus, on average, for the same gene, one replicate measurement can have as much as half of the signal intensity of the other replicate measurements (Figure 1B). At this level of experimental error, the two-fold difference that could be expected in expression levels related to MSCI would be undetectable.

### Statistical re-analysis of the larval testis expression genome-wide data

**Figure 2:**
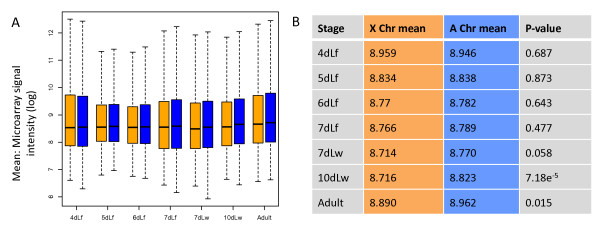
**X-linked and autosomal gene expression in developing testis**. (**A**) Box plot representing the distributions of the means of 10 replicates for approximately 14,000 *Drosophila *transcripts in each development stage [32]. (**B**) Mean values for X chromosome (X) and autosomes (A) in each development stage and their respective *P *values for the t-test of equality of the means. Colours code and abbreviations are described in the legend to Figure 1.

We also reproduced the genome-wide distribution of gene expression in testis of different developmental stages corresponding to their original Figure 2 in [32] (Figure 2A here). Statistical analysis could provide more information about the comparison between the expression of X chromosome and autosomal as opposed to the visual inspection done by Mikhaylova and Nurminsky [32]. The box plots of the chromosomes distribution of gene expression clearly show that the mean of X-linked genes was consistently lower compared to autosomes (Figure 2A). Indeed, except for the first larval development phase (fourth day, second instar), the X chromosome was consistently lower in terms of mean expression than autosomal expression (Figure 2B). This biased difference towards lower X-expression is against the random expectation that the two types of chromosomes have no differences in expression. More significantly, in the last three phases of later development (wandering larva through adult), the expression from the X chromosome was statistically lower than the autosomal expression from a marginal level to significant levels (*P *= 0.058, *P *= 7.18e^-5 ^and *P *= 0.015, respectively, t-test, Figure 2B). This experiment was conducted at 18°C (previous experiments were performed at 25°C), which extends the larval developmental time to 10 days and probably delays the entire timeline of the first wave of germline differentiation. The onset of pupation in this case occurs from the 11th to 12th day [32]. Therefore, it can be inferred that the change in experimental conditions resulted in the accumulation of increased numbers of meiotic cells during the latter stages of third instar larval testis development so that the effect of X inactivation became detectable as we just showed [32,34].

Contrary to Mikhaylova and Nurminsky's interpretations [32], our statistical analyses of their data suggest that the X chromosome expression level is lower than the expression level of autosomes during most stages of larval spermatogenesis, which is consistent with the expectations of MSCI. However, it should be pointed out that it is possible that the large experimental error, as we found above, may have compromised the power to detect two-fold difference of expression between chromosomes as expected by MSCI or the statistical signal detected from MSCI would be even greater.

### No general paucity of tissue-specific gene expression on the Drosophila X chromosome

**Figure 3:**
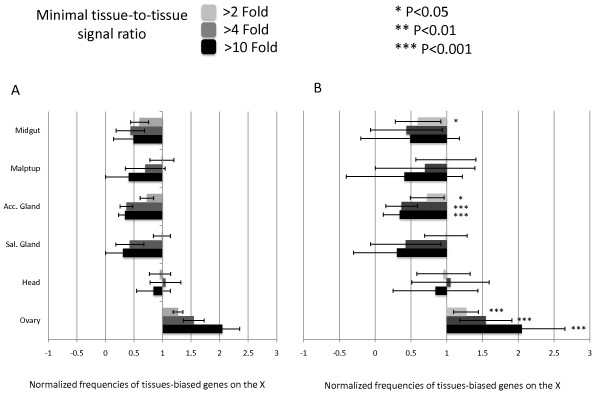
**Confidence intervals for normalized frequencies of tissue-biased genes on the X chromosome**. As in Figure 4 of [32], the ratio of microarray signal intensities observed between the tissue sample indicated at the left including midgut, malpigian tubule, accessory gland, salivary gland, head, and ovary was used as a measure of tissue bias. The columns correspond to the frequencies of the genes on the X chromosome normalized against the genome averages, and are shown for the genes with at least two-fold, five-fold, and ten-fold expression bias toward indicated tissues. Confidence intervals (bar) of 70% (**A**) and 95% (**B**) are shown for one and two standard error, respectively. Significant deviations (Chi-square with Yates correction) are indicated in (B) for datasets with minimal tissue-to-tissue signal ratio greater than two-, five-, and ten-fold (***P < 0.001; **P < 0.01, *P < 0.05).

**Figure 4:**
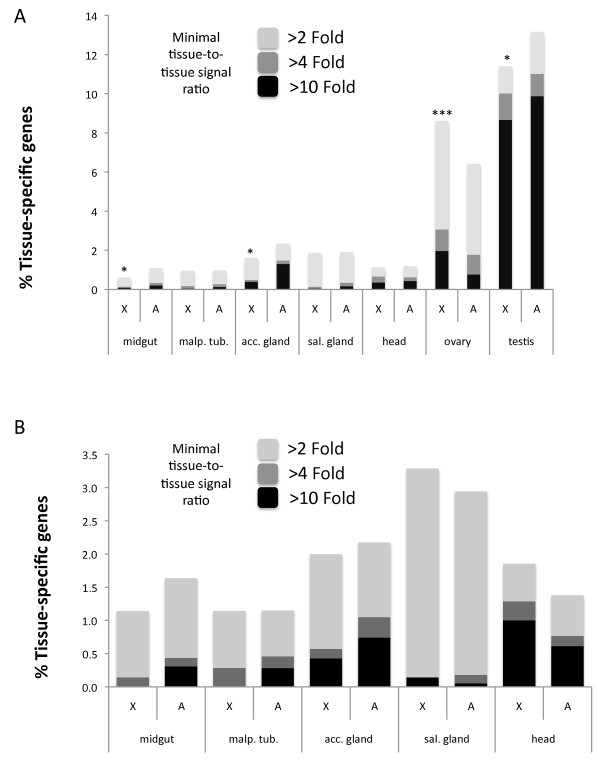
**Chromosomal distribution of tissue-biased genes**. Distribution of tissue-specific genes in the X chromosome and autosomes of *D. melanogaster*. (**A**) By an alternative way to present the data found in Figure 3B, we observed that only accessory gland-, mid-gut-, and testis-specific genes are significantly under-represented on the X chromosome. (**B**) The same chromosomal distribution analysis for tissue-specific genes was performed only with genes that are not testis- or ovary-biased expressed, that is, have the same expression in testis and ovaries. No differential chromosomal distribution is found for any of the analyzed dataset of tissue-specific genes. Significant deviations (Fisher's exact test or Chi-square with Yates correction) are indicated for tissue-specific datasets with minimal tissue-to-tissue signal ratio larger than two-fold (***P < 0.001; **P < 0.01, *P < 0.05).

The apparent lack of evidence for MSCI led Mikhaylova and Nurminsky [32] to ask whether the observed paucity of X-linked male-biased genes could be a simple consequence of a broader phenomenon that occurs for any gene exhibiting tissue-biased expression. To test this idea, they used Flyatlas [33] to assemble a candidate set of tissue-specific genes from numerous tissues and organs including the ovary and testis. To select candidates, they implemented the method of minimal tissue-to-tissue signal ratio [32]. In other words, a given gene is consider to be tissue-specific if its signal from Flyatlas [33] microarray is at least two times larger than the signals from all other tissues in the analysis (see Methods for details). They then analyzed the chromosomal distribution of these tissue-specific gene sets for differences between the X chromosome and the autosomes. They found that the proportions of over-expressed genes were under-represented in the X chromosome (Figure 3A, re-plotted, from [32]). In their original analysis [32], performed without statistical tests, revealed that almost all tissue-specific genes were under-represented in the X chromosome (Figure 4 in [32]). The only exception was for ovary-biased genes that were over-represented in the X chromosome, consistent with previous reports and in agreement with sexual antagonistic selection [1,15,16]. Our re-analysis of these data (Figure 3A) assessed the statistical significance of the chromosomal distributions using the 2 × 2 contingency tables. It is clear from Figure 3B that genes with biased expression in sex-specific tissues (accessory gland and ovaries Figure 3B; testis, Figure 4A) are under-/over-represented on the X chromosome. Mikhaylova and Nurminsky's [32] analysis was based on one standard error of the mean to compare the X chromosome and autosomal distributions of genes (Figure 3A). One SE interval covers only around 70% of the distribution and therefore is not able to accurately detect the overlaps between distributions. A re-plot of their data (Figure 4 in [32]) using two standard errors (Figure 3B) and therefore including the 95% confidence intervals supports the conclusion that mostly sex-specific tissues have a significantly skewed chromosomal distribution (see calculations in Additional file 3). The only exception for this rule is the dataset of midgut-specific genes. Normally, statistical tests that compare sampled means take into account the 95% confidence interval calculated for the correspondent parametric mean. That explains why our contingency table tests generally agrees with results generated with two standard errors measurements.

**Figure 5:**
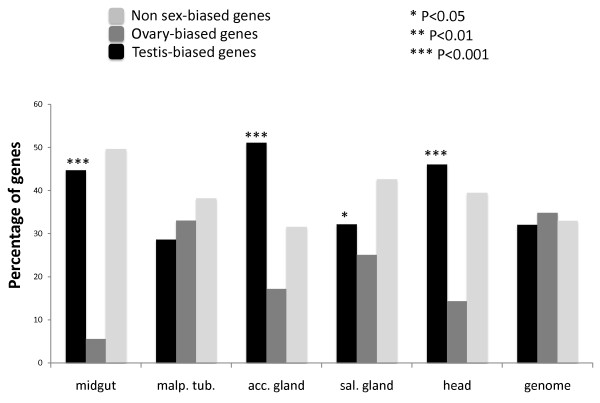
**Distribution of sex-bias biased genes in tissue-specific datasets**. Proportion of testis- and ovary-biased genes found in each tissue-specific gene dataset. For instances, testis-biased expression is defined for genes with signal ratio between testis and ovary larger than two. We tested if the distribution of testis-biased genes as compared to that of the ovary-biased genes were different between the tissue-specific dataset and the entire genome. Significant deviations from genomic proportions (Chi-square with Yates correction) are indicated for tissue-specific datasets with minimal tissue-to-tissue signal ratio larger than twofold (***P < 0.001; **P < 0.01, *P < 0.05).

Moreover, we note that these tissue-specific expression results used by Mihaylova and Nurminsky [32] were enriched with testis-biased genes. Many of the genes included for 'tissue-specific' expression were also expressed in testis at elevated levels (as compared to ovaries; Figure 5, Additional file 4). The method of minimal tissue-to-tissue signal ratio allows such data scenario [32]. For example, the gene CG7194 is midgut-specific (microarray average signal value = 629), but is higher expressed in testis than in ovaries (196 *vs*. 34, respectively, Additional File 4 under Oligo '1631098_at'). We found that all tissues analyzed, except for malpigian tubules, are significantly enriched with testis-biased genes where many of them reach testis and ovary expression differences greater than 10-fold (Figure 5).

It is therefore logical to assume that propensity of tissue-specific genes to be testis-biased explains the apparent generalized under-representation on the X chromosome [1,4,5]. To test this idea, we examined the chromosomal distribution of tissue-specific genes after removal of testis- and ovary-biased genes (see Methods). We found no significant under-representation of X-linked genes among all tissue types including the male specific accessory gland (Figure 4B; Figure 4A is a modified re-plot of the same data in Figure 3B). Indeed, although not statistically significant, we found that the salivary gland- and head-specific genes were more frequently found in the X chromosome (Figure 4B). In other words, following removal of confounding effects of correlated gene expression amongst tissue types, there is no statistical evidence in support of a general non-random chromosomal distribution of X-linked tissue-specific genes. The only two *Drosophila *tissues with skewed chromosomal distribution are testis and ovaries, which are enriched on the autosomes and on the X chromosome, respectively.

**Figure 6:**
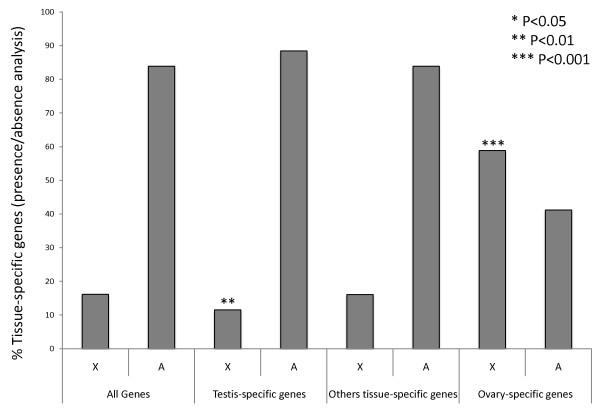
**Chromosomal distribution of tissue-specific genes present only in one single tissue**. Chromosomal distribution of our own dataset of tissue-specific genes. We required those to be present in a single tissue but completely absent in all other tissues. Testis- and ovary- specific genes are respectively under- and over- represented in the X chromosome (Chi-square test with Yates correction: *P *= 0.0082, *n *= 468; *P *= 0.0001, *n *= 17, respectively). Other tissue-specific genes were polled together and are not under-represented in the X chromosome (Chi-square test with Yates correction: *P *= 0.97, *n *= 118). The sample sizes for individual sets of tissue-specific genes are: accessory glands = 10; head = 37; brain = 20; larval hindgut = 26; larval midgut = 8. Hindgut, midgut, adult carcass, crop, salivary gland, thoracicoabdominal ganglion and larval and adult tubules < 5 (each). Except for brain-specific genes that are over-represented in the X chromosome (Chi-square test with Yates correction: *P *= 0.01), no other tissue show non-random chromosome distribution (for cases where *n *≥4). We analyzed only old genes since the chromosome X is known as enriched with new genes with testis-biased expression. Figure S3 in Additional file 1 show more details for differences between gene age and chromosomal distribution of testis-specific genes.

To further test if tissue-specific genes are non-randomly distributed between the X chromosome and the autosomes, we used an independent method to select tissue-specific genes. Genes present in one single tissue but completely absent in all other tissues defined our own dataset of tissue-specific genes (FlyAtlas [33] microarray experiments absence and presence calls, see Methods for details). Our findings remain the same: no other tissue-specific genes beside those expressed in testis and ovaries are differently distributed between the X chromosome and the autosomes (Figure 6).

We note that female-biased genes are also more expressed in other tissues than male-biased genes (Figure S2, Additional File 1) [1,37], a result that could substantially account for the trend found by Mihaylova and Nurminsky [32]. For instance, female-biased genes tend towards housekeeping genes and therefore less likely to be over expressed in a single tissue than in all others.

## Discussion

MSCI has been enthusiastically discussed and debated in the *Drosophila *literature for four decades [20,24,25,29-32]. On one hand, evidence for the existence of MSCI in *Drosophila *has been supported by a variety of experimental approaches [24,25,29,38], consistent with the conclusions drawn nearly 40 years ago using chromosomal translocations [20]. Insertions of the testis-specific promoter from *ocnus *gene were performed for different regions of the genome [24,25]. The X-linked insertions showed significant and consistent lower expression than autosomal ones [24] (here considered as evidence I). These results are in agreement with the MSCI model and rules out the possibility of a meiotic-specific lack of dosage compensation since only heterozygous insertions were analyzed for all chromosomes [24]. Reduced expression was also observed using insertions into several regions of the X chromosome suggesting that it is unlikely that large proportions of the chromosome escapes MSCI [29]. Independent supporting evidence comes from our stage-specific expression analyses of spermatogenesis [25] that revealed statistical evidence for down-regulation of the X chromosome during meiosis (evidence II). In addition, a recently published study [38] analyzed expression data from *bag-of-*marbles (bam) mutant testes [39]. bam mutations block entry into meiosis and result in overgrowth of primary spermatocytes [40]. This recent study showed a significant reduction in X chromosome expression in wild-type testes compared to bam mutant testes [38], a result entirely consistent with MSCI (evidence III). Consistent results were found also in [31].

Two recently published papers however fail to find evidence to support MSCI [31,32]. Meiklejohn and colleagues [31] re-analyzed and provided further data on spermatogenic stage-specific expression (evidence II) and found that the mitotic stage already presents significant down-regulation of the X chromosome. The authors claim that such pattern of X chromosome-specific down-regulation is not consistent with MSCI as it occurs prior to meiosis and they hypothesize that another unrecognized mechanism regulates X chromosome expression during spermatogenesis [31]. However, an important fact was neglected in their analysis. The dissections of the mitotic cells from intact testis are limited due to the mixed distribution between spermatogenic phases [25]. It is not yet technically possible to obtain pure mitotic cells from wild-type *Drosophila *testes [25] and it follows that these samples were contaminated with meiotic cells. Therefore, a much simpler interpretation is that the mitotic sample analyzed contains an unknown but potentially substantial proportion of meiotic cells that would reduce the expression levels of X-linked genes due to MSCI. The consequence of this confounding issue would be to create a misimpression that the expression pattern of down-regulation of the X chromosome occurs earlier during the mitotic stage of spermatogenesis [31]. To account for these empirical limitations of cellular composition on the observed expression differences between the X and autosomes, we developed a Bayesian statistical framework based on the relative difference between the spermatogenesis stages (mitosis compared to meiosis) and with the sensitivity to detect down regulation on the X-chromosome during the meiotic phase [25]. Nonetheless, both studies [25,31] agree that some down-regulation of the X chromosome occurs during spermatogenesis.

However, Mikhaylova and Nurminsky's study [32] using larval testis expression profiling found no differential expression between autosomes and X chromosomes in testis from different larval development stages [32]. As those testes contain mostly meiotic cells, MSCI was ruled out as a possible process [32]. Here we showed that the low correlation within the replicates present in each of the developmental testis stage compromised the genome-wide analysis based on replicate averages done by Mikhaylova and Nurminsky [32]. Nevertheless, (small) reduction in expression of the X, consistent with MSCI, was still detectable in the later stages of the testis development.

An extension of the debate about MSCI is the non-random chromosomal distribution of male-biased genes. MSCI has been proposed as one of the driving forces responsible for the paucity of testis-biased genes on the *Drosophila *X chromosome found by several different studies [1,3-5]. Mikhaylova and Nurminsky [32], however, have shown that the X-chromosome skewed pattern is not an exclusive feature of testis-biased genes, but instead is a general property of all tissue-specific genes. Based on that, they concluded that selective forces such as sexual antagonism and MSCI could not account for the observed chromosomal distribution [32]. Our re-examination of Mikhaylova and Nurminsky's study [32] revealed that their dataset of tissue-specific genes are actually enriched with testis-biased genes. We showed by several approaches that tissue-specific genes datasets, uncontaminated by testis-biased genes, show no sign of skewed chromosomal distribution patterns.

Our re-analysis of Mikhaylova and Nurminsky's data has clear consequences to the field. First, the under-representation of tissue-biased expression on the X chromosome is linked to *Drosophila *reproduction, and is predominantly only found for testis-biased/specific genes. Therefore, their hypothesis that the X chromosome provides an inferior environment for any type of tissue-specialized genes is not supported [32]. The same argument is true for their complementary experiments using chromatin-binding proteins presented in the same study [32] as they were based primarily on tissue-biased genes enriched in testis-expressed genes.

## Conclusions

Through the re-analysis and re-examination of Mikhaylova and Nurminsky [32], we found that the study, which presents evidence against the MSCI model, could not support their major findings. Moreover, all tissue-specific genes, except for those specifically expressed on the testes or the ovaries, are randomly distributed on the chromosomes.

Microarray expression data is difficult to collect and analyze, and we hope that the re-analyses of this study, whose conclusions are already being cited ([31,41]), will help re-center the field by providing a very rigorous treatment of the data used. Table 2 displays detailed description of the evidence supporting and refuting the existence of MSCI and its role as a driving force for the chromosomal distribution of male-biased genes. Our primary concern is to point out that the data presented in Mikhaylova and Nurminsky study [32] can be used neither as evidence against MSCI nor to support their claim of general under-representation on the X chromosome of tissue-specific genes. Therefore, after the re-analysis of Mikhaylova and Nurminsky's data [32], we find no reason to alter or reject the prevailing hypotheses of MSCI, sexual antagonism, meiotic drive, or dosage compensation [2,15-23].

**Table 2:** Controversies regarding meiotic sex chromosome inactivation (MSCI) and the chromosomal distribution of male-biased genes in *Drosophila*

	MSCI	**(1) X → A retrogenes with testis biased expression **[2]	**(2) Under-representation of male/testis-biased genes on the X **[1]
MSCI as driving force (**pros**/cons)	**Down-regulation of testis-specific insertions in the X**. Use a single promoter [24,29]. **Down-regulation of X in meiosis**. Use mixture of cells [25]. **Down-regulation of X in wild-type testis as opposed to bam mutant testis **[38]. bam mutant also show small degree of down regulation [31].	**Complementary expression in meiosis for X → A Retrogene **[25].	**Under-representation of male-meiotic expressed genes in the X **[25].
MSCI NOT as driving force **(pros/**cons)	**No global down regulation of the X chromosome in developing testis **[32]. No statistical support^a^.	**Retrogenes with general female or unbiased expression **[43]. No expression data support.	**General tissue-specific under-representation on the X **[32]. Tissue-specific genes are enriched with testis-biased genes^a^.

^a^Shown in the current work.

Supportive **(pros) **or conflicting (cons) data related to MSCI and its role on the chromosomal distribution of male-biased genes proposed through two observations: (1) X → A retrogenes with testis biased expression; (2) Under-representation of male/testis-biased genes on the X. Details on our findings of no expression data support for female-biased or unbiased expression of the retrogenes are presented in Vibranovski, Zhang, Kemkemer, Lopes, Karr and Long: Segmental dataset and whole body expression data do not support the hypothesis that non-random movement is an intrinsic property of Drosophila retrogenes, submitted.

## Methods

### Testis development data and analysis

Normalized data of testis development stages were obtained and parsed out from processed files available in Array express submission E-MEXP-1980 [32]. Normalized expression values were parsed out according to P-MTAB-2894 protocol for bioassay data transformation: M = R-G (log fold change); A = (R+G)/2 (average intensity) where R and G are normalized log transformed red and green channel intensities. Null values for A (average intensity) were excluded from the statistical analyses. Chromosomal locations were obtained by cross-linking the CG information of the processed files to Drosophila genome release 5.1 downloaded from Flybase [35]. Statistical parameters such as means, standard deviations and correlations were calculated using Additional File 2 which were plotted in R. The source of the variance corresponding to experimental error (s^2 ^within replicates) was calculated using the sum of squares method for nested Anova according to [42].

### Tissue-specific data and analysis

The first dataset of tissue-specific genes was obtained as described in [32]. Briefly, using gene expression data available in FlyAtlas [33], microarray signals derived from a specific tissue were compared to similar signal derived from a panel of tissues as shown in Additional File 4. Tissue-specific genes were selected when the minimal tissue-to-tissue signal ratio across the entire panel were > 2, > 5, and > 10.

The second dataset of tissue-specific genes was obtained using FlyAtlas [33] information of presence and absence in microarray expression data. Tissue-specific genes were selected when present, for a single tissue, in four microarray experimental replicates but absent in all replicates of all other tissues (see the considered panel of tissues in [32]). We analyzed only genes that originated before the split between the *Sophophora *and *Drosophila *subgenus to avoid the confounding effect from new genes that are usually enriched in the X chromosome if testis-biased expressed (as described in [3]). The age analysis was performing by crosslinking 'CG' numbers with age information in [3].

Sex-biased and unbiased genes were selected according to [1]. For example, genes were considered male-biased whenever the signal ratio between testis and ovary microarray intensities was larger than two. Only probes with CG and chromosome location information were used in our analyses, but major results are reproducible using all probes with chromosome location. Information was obtained by cross-linking the 'Oligo' information from Flyatlas [33] downloadable file (20090519all.txt) and 'Probe Set ID' from Affymetrix annotation file (drosophila_2.na23.annot.csv). We used only 'Probe Set IDs' with a unique alignment in the genome. Significances of Fisher exact test were calculated in R whenever the total sample size was smaller than 5,000 cases; otherwise chi-square tests with Yates correction were performed. First, differences in the proportions of X-linked and autosomal genes in tissue-specific sets and in the rest of the genome were assessed (Figures 3, 4, and 6). Second, male-biased enrichment was called by comparing proportions of testis-biased and non-testis-biased genes for each tissue-specific group against the same proportion on the rest of the genome (Figure 5) [1,5].

## Authors' contributions

MDV, TLK, and ML conceived the study. MDV and YEZ performed the computational experiments. MDV, YEZ, and CK analyzed the data. MDV and HFL performed the statistical analyses. MDV, TLK, and ML collated, assembled and, with assistance and approval of all authors, wrote the manuscript.

## Supplementary Material

Additional file 1 **Figures S1-S3**. Click here for file

Additional file 2 **Normalized testis developmental expression**. Normalized expression (log_2 _based) from each testis developmental stage [32] for each *Drosophila *transcript with corresponding chromosomal location in xls format ('CG identification: transcript number'). Click here for file

Additional file 3 **Input for re-plot of Figure 4 in**[32]. Re-plots are shown in Figure 3A and 3B. Click here for file

Additional file 4 **Tissue specific gene dataset**. List of tissue-specific genes obtained through Flyatlas [33] expression (Methods and [32]). Each excel sheet corresponds to one analyzed adult tissue: midgut, malpigian tubules, accessory glands, salivary gland, head, ovary, and testis. Minimal fold between one tissue against all other tissues analyzed is shown for < 2, < 5, and < 10. Sex-bias is shown for all genes as: M, male-biased; F, female-biased; and U, unbiased. Click here for file
