# Supplementary material for: Re-analysis of the larval testis data on meiotic sex chromosome inactivation revealed evidence for tissue-specific gene expression related to the drosophila X chromosome
Source: BMC Biol. 2012 Jun 12;10:49. doi: 10.1186/1741-7007-10-49 (PMC3391172; doi:10.1186/1741-7007-10-49)
Supplement: Additional file 1 — Figures S1-S3. [file 1741-7007-10-49-S1.DOC]

**
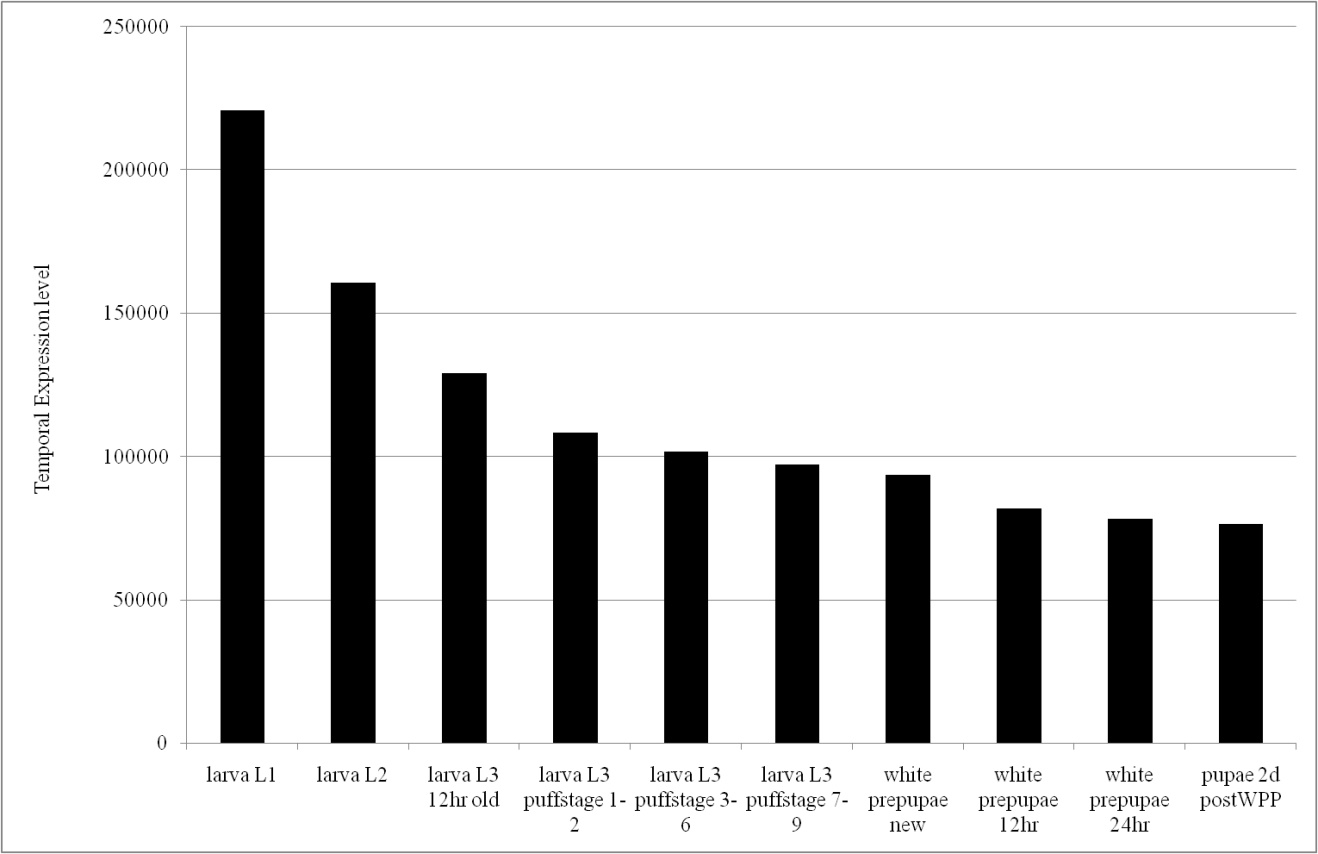
**

**Figure S1.** Temporal expression profile of the *rp49* gene: first instar larvae to pupae stage. The X axis shows the different development stages and Y axis shows the expression level, according to Flybase [1]. The expression was measured in whole larva, pupae or flies. The decrease in expression is approximately in the range of 2-3 fold difference comparing the first stages of larvae development and the onset of pupation. Assuming that the temporal expression of the *rp49* transcript is similar between whole flies and the temporal expression of *rp49* transcript in the testis, reasonable for a housekeeping gene, the observed dramatic increased expression of the eight X-linked genes and the 18 autosomal genes is less dramatic than reported [2].


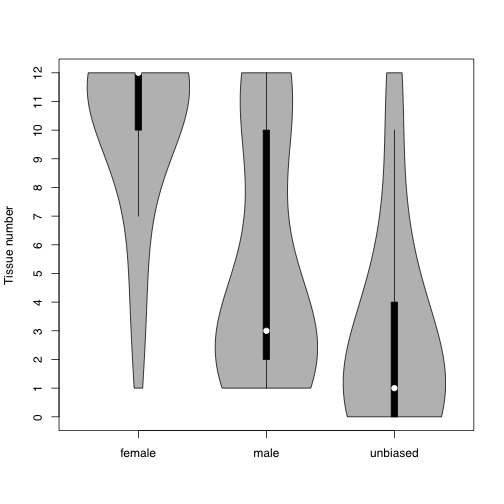


**Figure S2.** Distribution of genes found to be present in a given number of tissues in Flyatlas database [3]. The X-axis represents three categories, while Y-axis means the number of tissues where a given gene is transcribed. For the violin plot, the white circle marks the median. The thick black bar represents the 25% and 75% percentiles, while the shape shows the distribution density. The microarray processing procedure follows [4]. Briefly, we used the GCRMA package to adjust the background intensity, normalize and summarize the expression, the Affymetrix package to call the presence or absence of each gene, the Limma package to assess whether genes show differential expression between testis and ovary.


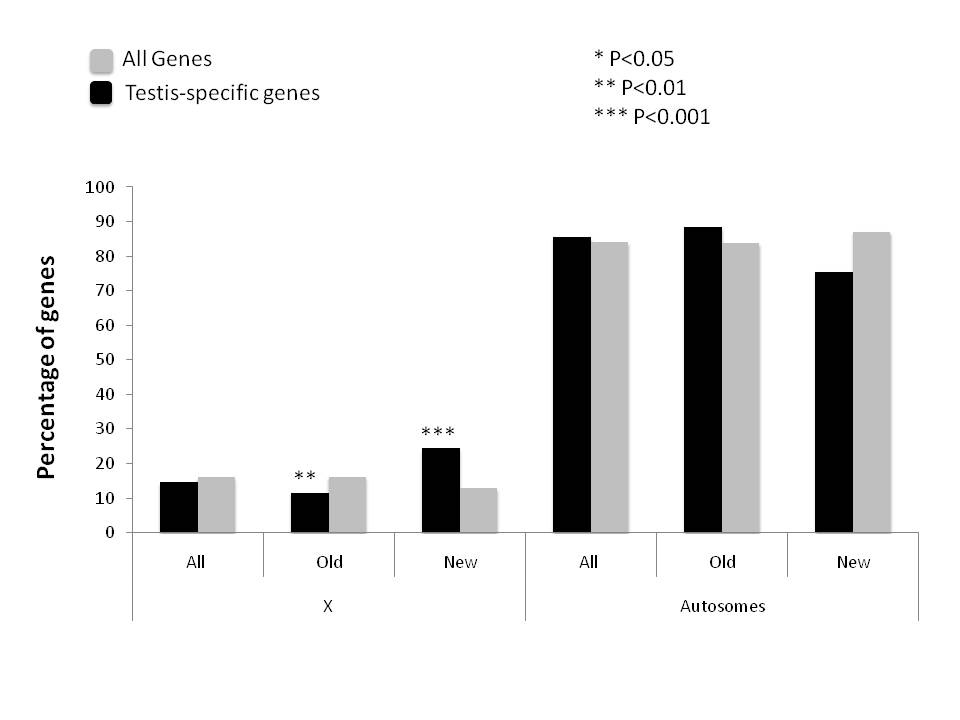


**Figure S3.** Chromosomal distribution of testis-specific genes according to their evolutionary age. Testis-specific genes were defined by those expressed only in testis but completely absent in all other analyzed tissues. New and old genes were defined according to [4]. At first glance, testis-specific genes (All) are randomly distributed among X and autosomes (Chi square test with Yates correction, p=0.33). However, testis-specific genes at least as old as the split between Sophophora and Drosophila subgenus are under-represented in the X chromosome (Chi square test with Yates correction, p=0.006), while new testis-specific genes are found in excess in the X chromosome. Those results are in agreement with previous findings that the chromosomal distribution of male-biased genes depends on their age [4]. Different than testis-biased genes (those more expressed in testis than in ovaries), testis-specific genes in general are not under-represented in the X chromosome because this dataset is enriched with new genes (23% vs. 4%, Chi square test with Yates correction, p<0.001).

References

1. Tweedie S, Ashburner M, Falls K, Leyland P, McQuilton P, Marygold S, Millburn G, Osumi-Sutherland D, Schroeder A, Seal R, Zhang H, and The FlyBase Consortium**: FlyBase: enhancing Drosophila Gene Ontology annotations.** *Nucleic Acids Research* 2009, **37:**D555-D559.
2. Mikhaylova LM, Nurminsky DI: **Lack of global meiotic sex chromosome inactivation, and paucity of tissue-specific gene expression on the Drosophila X chromosome.** *BMC Biol.* 2011, **9:**29.
3. Chintapalli VR, Wang J, Dow JA: **Using FlyAtlas to identify better *Drosophila melanogaster* models of human disease.** *Nat Genet* 2007, **39:**715-720.
4. Zhang YE, Vibranovski MD, Krinsky BH, Long M: **Age-dependent chromosomal distribution of male-biased genes in Drosophila.** *Genome Res* 2010, **20:**1526-1533.
